# Inhibition of ARC decreases the survival of HEI-OC-1 cells after neomycin damage *in vitro*

**DOI:** 10.18632/oncotarget.11336

**Published:** 2016-08-20

**Authors:** Ming Guan, Qiaojun Fang, Zuhong He, Yong Li, Fuping Qian, Xiaoyun Qian, Ling Lu, Xiaoli Zhang, Dingding Liu, Jieyu Qi, Shasha Zhang, Mingliang Tang, Xia Gao, Renjie Chai

**Affiliations:** ^1^ Department of Otolaryngology, The Affiliated Hangzhou Hospital of Nanjing Medical University, Hangzhou 310006, China; ^2^ Department of Otolaryngology, Hangzhou First People's Hospital, Hangzhou 310006, China; ^3^ Department of Otolaryngology, Nanjing Drum Tower Hospital, Clinical College of Nanjing Medical University, Nanjing 210008, China; ^4^ MOE Key Laboratory of Developmental Genes and Human Disease, State Key Laboratory of Bioelectronics, Institute of Life Sciences, Southeast University, Nanjing 210096, China; ^5^ Co-Innovation Center of Neuroregeneration, Nantong University, Nantong 226001, China; ^6^ Department of Otolaryngology, Nanjing Drum Tower Hospital, The Affiliated Hospital of Nanjing University Medical School, Nanjing 210008, China

**Keywords:** cochlea, hair cell, apoptosis, reactive oxygen species, mitochondrial function

## Abstract

Hearing loss is a common sensory disorder mainly caused by the loss of hair cells (HCs). Noise, aging, and ototoxic drugs can all induce apoptosis in HCs. Apoptosis repressor with caspase recruitment domain(ARC) is a key factor in apoptosis that inhibits both intrinsic and extrinsic apoptosis pathways; however, there have been no reports on the role of ARC in HC loss in the inner ear. In this study, we used House Ear Institute Organ of Corti 1 (HEI-OC-1) cells, which is a cochlear hair-cell-like cell line, to investigate the role of ARC in aminoglycoside-induced HC loss. ARC was expressed in the cochlear HCs as well as in the HEI-OC-1 cells, but not in the supporting cells, and the expression level of ARC in HCs was decreased after neomycin injury in both cochlear HCs and HEI-OC-1 cells, suggesting that reduced levels of ARC might correlate with neomycin-induced HC loss. We inhibited ARC expression using siRNA and found that this significantly increased the sensitivity of HEI-OC-1 cells to neomycin toxicity. Finally, we found that ARC inhibition increased the expression of pro-apoptotic factors, decreased the mitochondrial membrane potential, and increased the level of reactive oxygen species (ROS) after neomycin injury, suggesting that ARC inhibits cell death and apoptosis in HEI-OC-1 cells by controlling mitochondrial function and ROS accumulation. Thus the endogenous anti-apoptotic factor ARC might be a new therapeutic target for the prevention of aminoglycoside-induced HC loss.

## INTRODUCTION

Sensorineural hearing loss is usually permanent because the human cochlea contains only about 5,000 hair cells (HCs), and these are terminally differentiated cells with very little capacity to regenerate after birth. The main causes of such hearing loss are noise, aging, and ototoxic drugs, all of which can induce apoptosis in HCs. Aminoglycoside-induced HC damage is one of the major causes of HC death [1], and several studies have reported that aminoglycoside treatment induces the intrinsic apoptosis of HCs through oxidative stress [2–6]. However, the genes regulating the ototoxic sensitivity of HCs are largely unknown, and the mechanisms involved in the ototoxic-sensitivity of HCs are not well understood.

Apoptosis repressor with caspase recruitment domain (ARC) is an important anti-apoptotic protein in both mitochondrial and death receptor apoptosis pathways [7]. ARC contains a caspase recruitment domain (CARD) region that is homologous to the CARDs of caspases and caspase adaptor proteins. Thus ARC might modulate apoptotic signaling by interacting with caspases or adaptor proteins. To our knowledge, ARC is predominantly expressed in post-mitotic cells such as cardiomyocytes, skeletal muscle cells, vascular smooth muscle, and neurons as well as in cochlear spiral ganglion neurons [7, 8]; but we have found no reports describing the expression and role of ARC in the inner ear sensorineural HCs. Similar to the cells described above, inner ear HCs are also post-mitotic, non-self-regenerative, and susceptible to aminoglycoside damage through apoptotic pathways, thus we hypothesized that the ARC protein might also be expressed in inner ear HCs and might protect HCs from aminoglycoside-induced damage. In this study, we took advantage of the HC-like House Ear Institute Organ of Corti 1 (HEI-OC-1) cell line to investigate the role of ARC in aminoglycoside-induced cell death. The HEI-OC-1 cell line has been used as a cochlear HC-like cell line in many studies, and these cells express several molecular markers of cochlear HCs, including calbindin, calmodulin, math1, myosin7a, and prestin [9–12].

In the present study, we show that ARC is expressed in the cochlear HCs and HEI-OC-1 cells and that inhibition of ARC by siRNA significantly increases the cells’ sensitivity to neomycin toxicity. Mechanistically, we revealed that ARC inhibition significantly promotes both intrinsic and extrinsic apoptotic factors by increasing mitochondrial dysfunction and ROS accumulation. Thus our study highlights the role of ARC in protecting HEI-OC-1 cells from neomycin-induced damage.

## RESULTS

### ARC is expressed in the cochlear HCs and HEI-OC-1 cells

**Figure 1:**
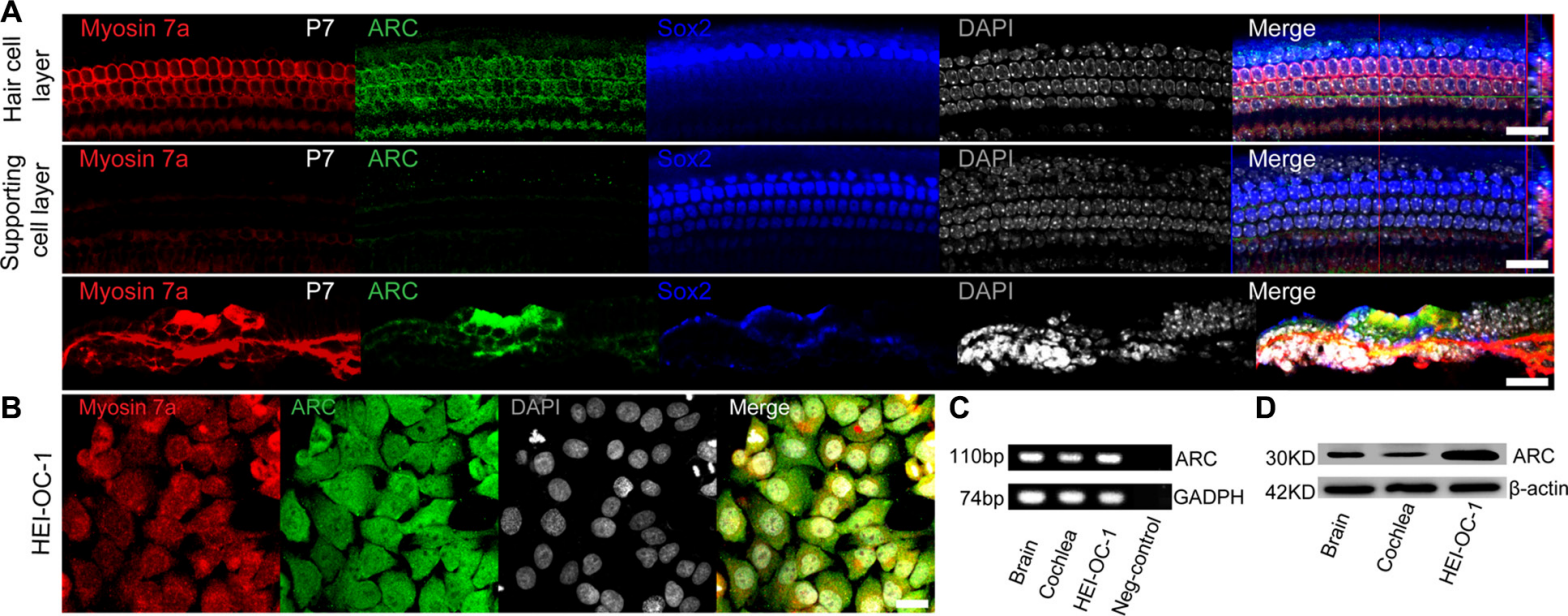
ARC was expressed in the cochlear HCs and HEI-OC-1 cells (**A**) Immunofluorescence staining showed that ARC was specifically expressed in the HCs, but not in the supporting cells of the organ of Corti in the P7 mice. Myosin 7a and Sox2 were used as markers for HCs and supporting cells, respectively. (**B**) Immunofluorescence staining showed that ARC was expressed in HEI-OC-1 cells. (**C**) RT-qPCR showed that ARC was expressed in the cochlea and in HEI-OC-1 cells. Brain samples were used as positive controls, and GAPDH served as a loading control in each lane. (**D**) Western blot showed that ARC was expressed in the cochlea and HEI-OC-1 cells. β-actin served as a loading control in each lane. Scale bars = 20 μm.

ARC has been reported to be expressed in cochlear spiral ganglion neurons [8]; but we are aware of no report on the expression and function of ARC in inner ear sensory HCs. In this study, RT-PCR and western blot data demonstrated that ARC was highly expressed in the postnatal day (P)7 mouse cochlea (Figure 1C and 1D). Immunohistochemistry further showed that ARC was specifically expressed in the HCs but not in the supporting cells of the P7 mouse organ of Corti (Figure 1A). Because most HC damage occurs in adults, we also investigated the expression of ARC in adult mice. Our results showed that ARC was also expressed in cochlear cells of P30 mice (Supplementary Figure S1).

The HEI-OC-1 cell line is a hair-cell-like cell line that is commonly used to study the damage and protection of HCs [9–12]. We used immunohistochemistry and RT-PCR to confirm that this cell line expresses several HC markers, including Myosin7a and Myosin6a, thus this cell line can serve as an HC-like cell line (Figure 1B, Supplementary Figure S2). Immunohistochemistry, RT-PCR, and western blot results demonstrated that ARC was also expressed in the HEI-OC-1 cells (Figure 1B–1D).

### ARC expression in HCs is decreased after neomycin injury

**Figure 2:**
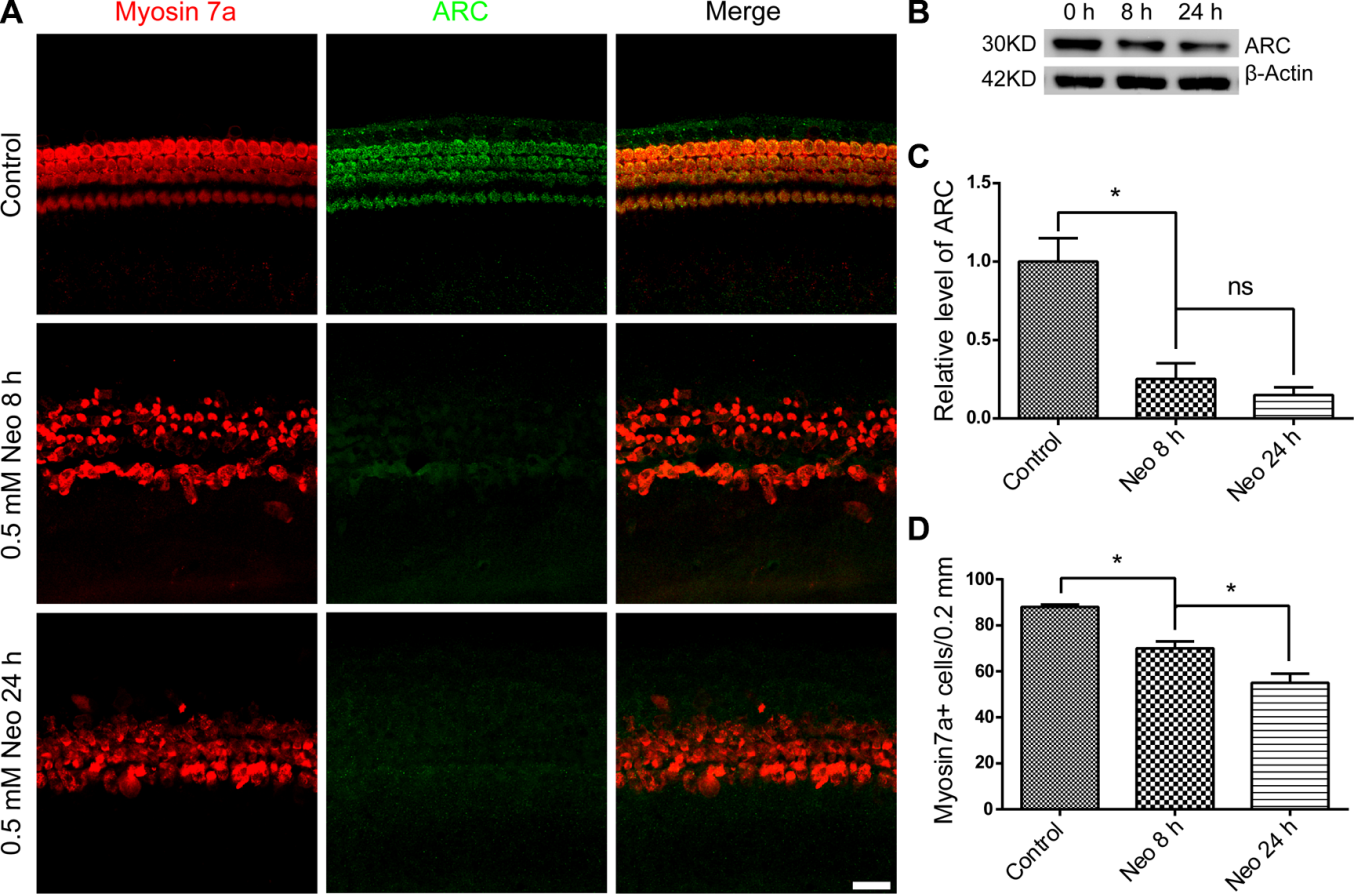
The ARC level in cochlear HCs decreased after neomycin injury (**A**) Immunofluorescence staining showed that ARC expression decreased during neomycin treatment. The cochlear sensory epithelium was dissected from P7 mice and cultured with 0.5 mM neomycin for 8 h or 24 h. After neomycin was removed, the tissues were cultured in serum-free medium for an additional 24 h. (**B** and **C**) The immunoblotting confirmed that the expression of ARC decreased during neomycin treatment. The protein loading was illustrated using an anti β-actin antibody. (**D**) Cochlear HC counts revealed a gradual loss of HCs, and this was much slower than the rate of reduction of the ARC protein level. Data are shown as mean ± S.D.*p < 0.05. Scale bars = 20 μm.

A previous study reported that the exposure of neonatal rat ventricular cardiomyocytes to doxorubicin resulted in the down-regulation of ARC and a subsequent increase in apoptosis [13]. To explore whether the ARC expression level in cochlear HCs was affected by neomycin, we treated the cultured cochleae with 0.5 mM neomycin for different times. We found that the HC number gradually decreased at 8 h and 24 h after neomycin treatment (Figure 2A and 2D) and that the expression of ARC in cochlear HCs rapidly decreased after neomycin treatment (Figure 2A–2C). Together, these results demonstrated that neomycin injury led to a rapid decrease in the expression of ARC in HCs, and this indicated that ARC might be involved in neomycin-induced HC apoptosis.

### The expression of ARC in HEI-OC-1 cells decreases after neomycin injury

To select the proper conditions to induce cell death in HEI-OC-1 cells, we exposed HEI-OC-1 cells to different doses of neomycin (1 mM to 20 mM) for different times (1 h to 24 h). We found that the viability of HEI-OC-1 cells decreased gradually with increasing neomycin doses and time, and about a 50%–60% of the HEI-OC-1 cells were dead after being treated with 10 mM neomycin for 24 h. This was the condition chosen for most of the further experiments investigating the detailed mechanism (Supplementary Figure S3).

**Figure 3:**
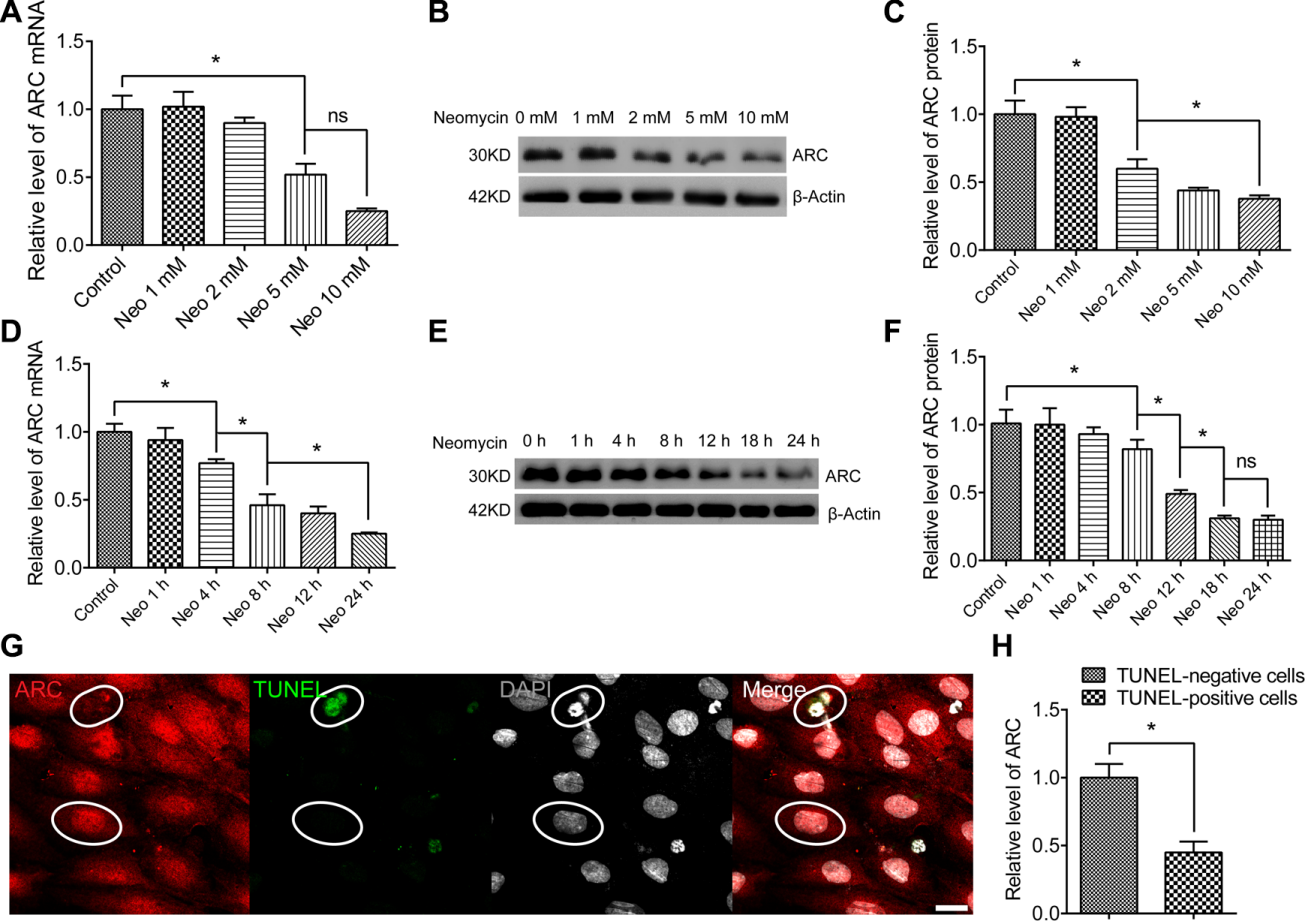
The ARC level in HEI-OC-1 cells decreased after neomycin injury The HEI-OC-1 cells were treated with 1 mM to 10 mM neomycin for 24 h, cultured in serum-free medium for an additional 24 h, then harvested for RT-qPCR and western blot. (**A**–**C**) The mRNA and protein levels of ARC in HEI-OC-1 cells decreased gradually with the increasing neomycin doses. (**D**–**F**) The mRNA and protein levels of ARC in HEI-OC-1 cells decreased with the increasing neomycin treatment time. (**G** and **H**) Immunofluorescence showed that the expression of ARC in TUNEL-positive cells was lower than in TUNEL-negative cells. The HEI-OC-1 cells were cultured with 10 mM neomycin for 24 h. TUNEL-positive cells were defined as apoptotic cells. Data are shown as mean ± S.D. *p < 0.05. Scale bars = 20 μm.

To further examine the expression of ARC in HEI-OC-1 cells after neomycin treatment, we treated the HEI-OC-1cells with 1 mM to 10 mM neomycin for 24 h. RT-qPCR and western blot results showed that the ARC mRNA and protein level decreased with increasing neomycin doses (Figure 3A–3C). Next we treated the HEI-OC-1 cells with 10 mM neomycin from 1 h to 24 h and found that the expression of ARC decreased with increasing time (Figure 3D–3F). We then treated the HEI-OC-1 cells with 10 mM neomycin for 24 h, and immunofluorescence data showed that ARC expression was significantly lower in TUNEL-positive apoptotic cells compared to TUNEL-negative cells (Figure 3G–3H), suggesting that the expression of ARC was significantly decreased in apoptotic cells. Together, these results demonstrated that ARC expression in HEI-OC-1 cells decreased in a dose- and time-dependent manner after neomycin injury and thus that ARC might be involved in neomycin-induced apoptosis in HEI-OC-1 cells.

### ARC inhibition with siRNA increases cell death and apoptosis in HEI-OC-1 cells after neomycin injury

**Figure 4:**
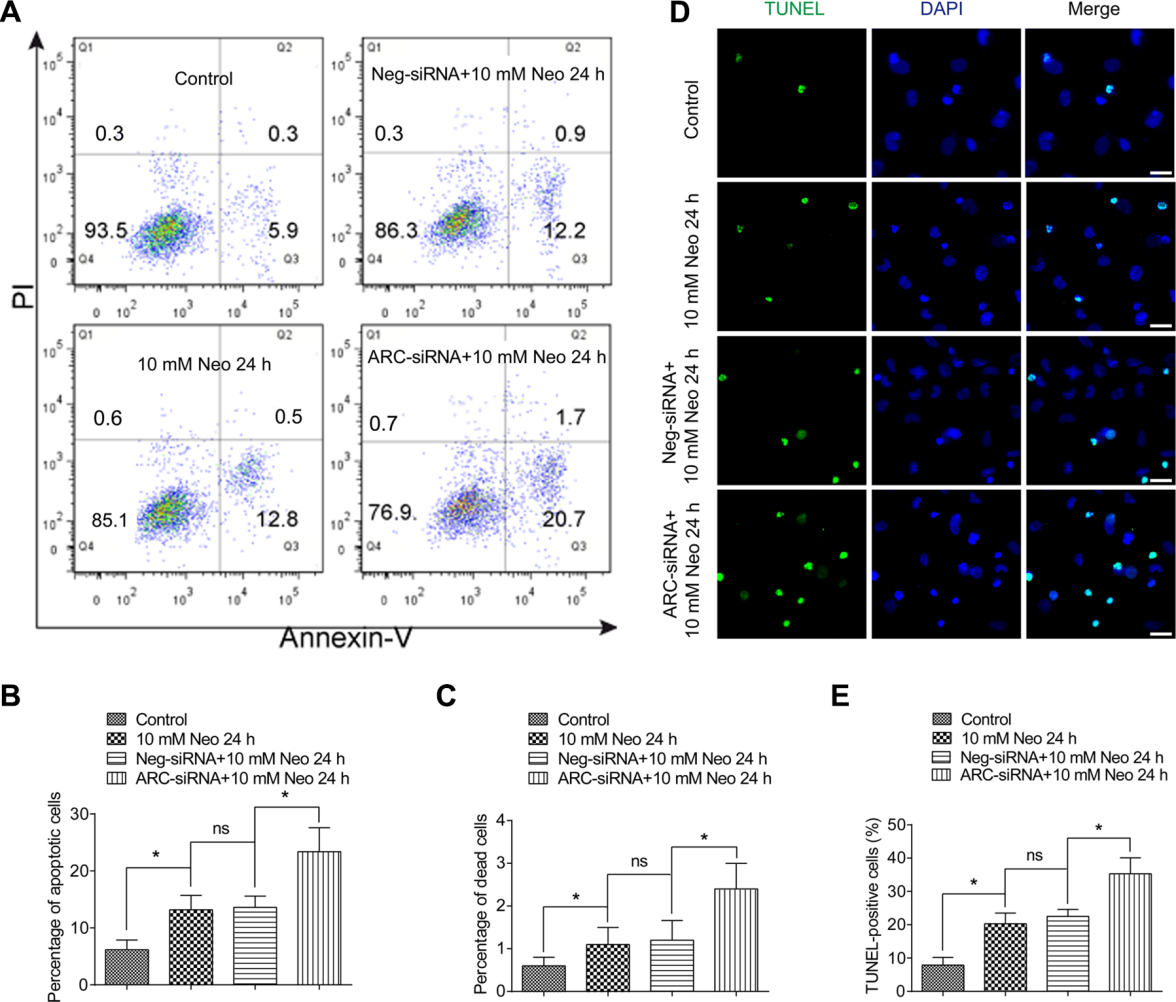
The percentages of both dead and apoptotic HEI-OC-1 cells increased after neomycin injury when ARC was inhibited by siRNA (**A**–**C**) Flow cytometry analysis showed that the percentages of both dead and apoptotic cells were significantly increased with 10 mM neomycin treatment for 24 h compared to the undamaged controls. The ARC-siRNA-transfected cells had significantly higher percentages of dead and apoptotic cells than the negative-siRNA-transfected controls after 10 mM neomycin treatment for 24 h. (**D** and **E**) TUNEL staining showed that the percentage of TUNEL-positive apoptotic cells increased in the ARC-siRNA-transfected groups compared with the negative-siRNA-transfected controls. Data are shown as mean ± S.D. *p < 0.05. Scale bars = 20 μm.

In order to investigate the role of ARC in the neomycin-induced cell death and apoptosis of HEI-OC-1 cells, we inhibited ARC with siRNA (the ARC-siRNA and negative-siRNA sequences are listed in Table 1). We transfected the HEI-OC-1 cell line with three individual ARC-siRNAs and a mixture of all three ARC-siRNAs. RT-qPCR, western blot, and immunofluorescence results showed that ARC expression was significantly reduced with the ARC-siRNA mixture (Supplementary Figure S4A–S4C), thus we used the mixture of three ARC-siRNA oligos for further experiments. First we tested whether knockdown of ARC itself would increase apoptosis and cell death in HEI-OC-1 cells without neomycin damage. The flow cytometry results showed that there was no significant difference in apoptosis or death of HEI-OC-1 cells after ARC inhibition without neomycin damage (Supplementary Figure S5A–S5C). Next we inhibited ARC with siRNA to determine the numbers of apoptotic and dead HEI-OC-1 cells after 10 mM neomycin treatment for 24 h (Figure 4A). We found that the percentages of both apoptotic and dead cells were significantly increased after neomycin treatment compared to the undamaged controls (Figure 4A–4C). Notably, after neomycin injury the cells transfected with ARC-siRNA had significantly greater percentages of apoptotic and dead cells than the controls transfected with negative-siRNA (Figure 4A–4C). To confirm this finding, we performed TUNEL staining to detect the apoptotic HEI-OC-1 cells after 10 mM neomycin treatment for 24 h. We found a significantly higher percentage of TUNEL-positive cells in the neomycin-treated groups compared with the undamaged controls (Figure 4D and 4E), and the ARC-siRNA-transfected groups had significantly higher percentages of TUNEL-positive cells after neomycin damage compared to the controls transfected with negative-siRNA (Figure 4D and 4E). We also determined the neomycin dose response curve and temporal response curve after ARC-siRNA transfection. The CCK-8 results showed that the cell survival ratio of ARC-siRNA-transfected groups decreased significantly faster compared to the controls transfected with negative siRNA when the dose and treatment time of neomycin increased (Supplementary Figure S6A and S6B). These results showed that ARC inhibition increased cell apoptosis and death in HEI-OC-1 cells after neomycin injury and indicated that ARC might protect HEI-OC-1 cells from neomycin ototoxicity.

**Table 1:** SiRNA sequences used in the experiments

siRNA	Forward sequence	Reverse sequence
ARC-siRNA 1	CGGAAACGGCUGGUAGAAATT	UUUCUACCAGCCGUUUCCGTT
ARC-siRNA 2	GAGUAUGAAGCCUUGGAUGTT	CAUCCAAGGCUUCAUACUCTT
ARC-siRNA 3	CCCAGCAAACUGUGAGCAUTT	AUGCUCACAGUUUGCUGGGTT
Negative siRNA	UUCUCCGAACGUGUCACGUTT	ACGUGACACGUUCGGAGAATT

### ARC inhibition with siRNA increases the expression of apoptotic factors in HEI-OC-1 cells after neomycin injury

**Figure 5:**
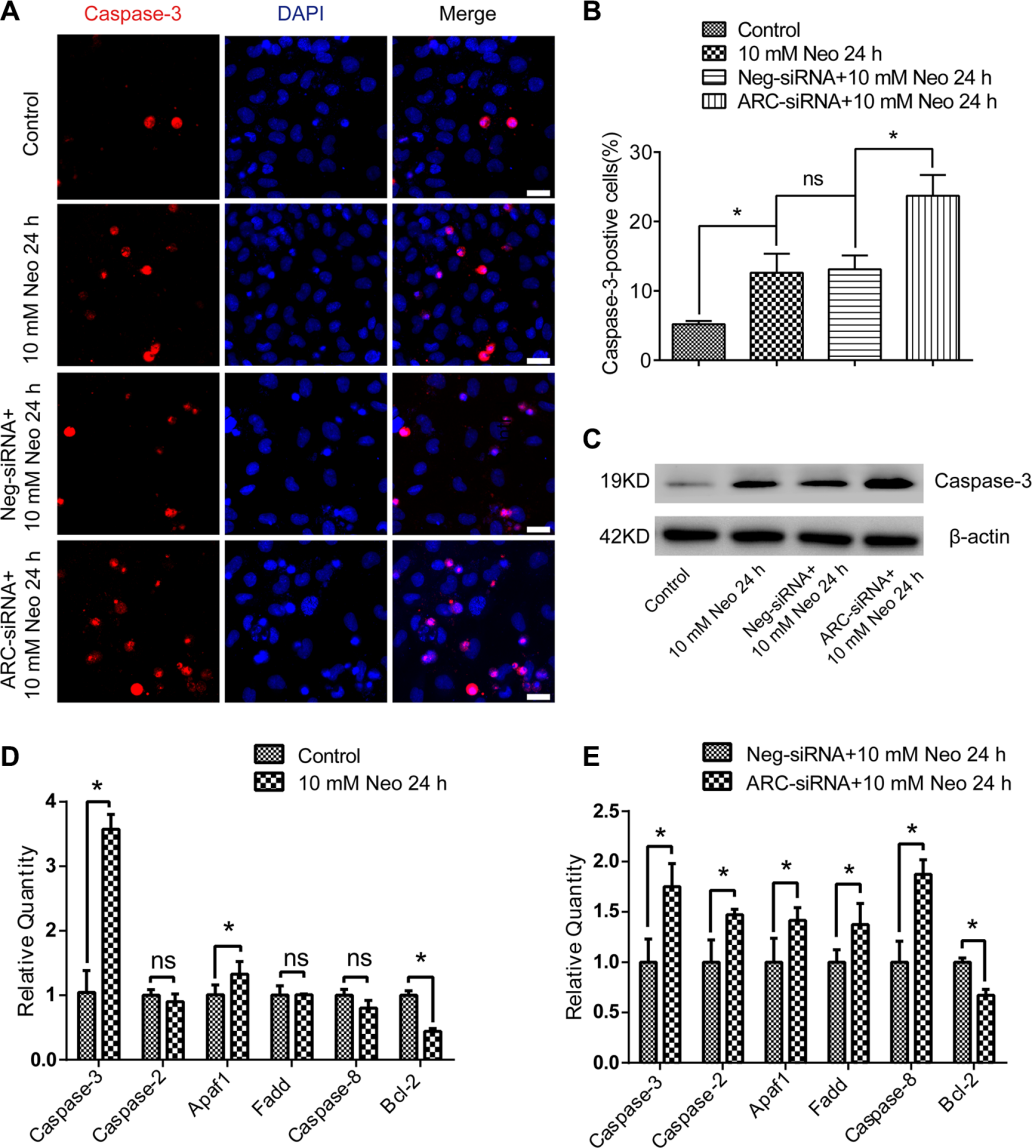
ARC inhibition increased the expression of proapoptotic factors in HEI-OC-1 cells after neomycin injury (**A** and **B**) Immunofluorescence showed that caspase-3-positive cells increased after 10 mM neomycin treatment for 24 h compared with the undamaged controls. Caspase-3-positive cells increased in the groups transfected with ARC-siRNA compared to the negative siRNA-transfected controls. (**C**) Western blot results confirmed that the expression of caspase-3 was higher in the neomycin-treated groups compared with the undamaged controls, while the ARC-siRNA transfected groups had significantly higher caspase-3 expression levels compared to the controls transfected with negative-siRNA. (**D**) RT-qPCR results showed that the expression of caspase-3 and Apaf1 were significantly increased and the anti-apoptotic factor Bcl-2 was significantly decreased, while the expression of the extrinsic apoptotic factors caspase-2, caspase-8, and Fadd were not significantly changed in neomycin-treated groups compared with the undamaged controls. (**E**) RT-qPCR results showed that the ARC-siRNA-transfected groups had significantly higher expression of both intrinsic and extrinsic apoptotic factors, while the anti-apoptotic factor Bcl-2 was significantly lower compared to the negative-siRNA-transfected controls. Data are shown as mean ± S.D. *p < 0.05. Scale bars = 20 μm.

Here we investigated the effects of ARC on the expression of pro-apoptotic and anti-apoptotic factors in HEI-OC-1 cells after neomycin injury. First, we used immunohistochemistry and western blot to evaluate the expression of cleaved caspase-3 in HEI-OC-1 cells after exposure to 10 mM neomycin for 24 h. We found a significantly higher percentage of caspase-3-positive cells and higher levels of caspase-3 expression in the neomycin-treated groups compared to the undamaged controls (Figure 5A–5D). In particular, the ARC-siRNA-transfected groups had a significantly higher percentage of caspase-3-positive cells and higher levels of caspase-3 expression compared to the controls transfected with negative siRNA (Figure 5A–5E). Furthermore, RT-qPCR results showed that the expression of the intrinsic apoptotic factor Apaf1 was significantly increased and the anti-apoptotic factor Bcl-2 was significantly decreased in neomycin-treated groups compared to the undamaged controls, while the expression of the extrinsic apoptotic factors caspase-2, caspase-8, and Fadd were not significantly different in the neomycin-treated groups (Figure 5D). In the ARC-siRNA-transfected groups, the expression levels of both intrinsic and extrinsic apoptotic factors were all significantly increased, and the anti-apoptotic factor Bcl-2 was significantly decreased, compared to the controls transfected with negative-siRNA (Figure 5E). Together, our results suggested that ARC is involved in neomycin-induced HEI-OC-1 apoptosis by inhibiting the expression of both intrinsic and extrinsic apoptotic factors.

### ARC inhibition with siRNA decreases the mitochondrial transmembrane potential of HEI-OC-1 cells after neomycin injury

**Figure 6:**
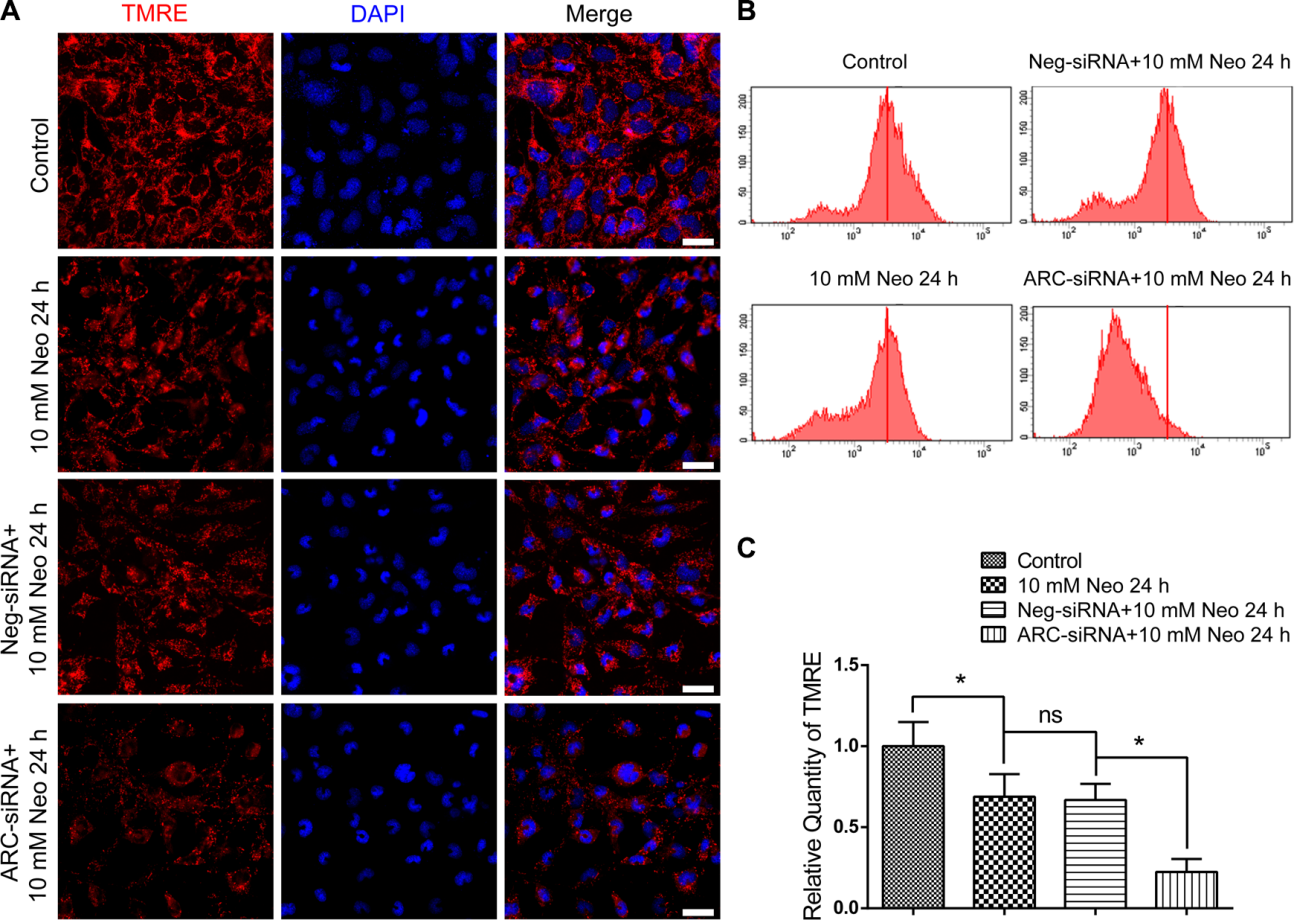
ARC inhibition decreased the MMP of HEI-OC-1 cells after neomycin injury (**A**) The immunofluorescence intensity of TMRE was decreased after 10 mM neomycin treatment for 24 h compared with the undamaged controls, and TMRE intensity in the ARC-siRNA-transfected groups was significantly reduced compared with the controls transfected with negative-siRNA. (**B** and **C**) Flow cytometry analysis showed that TMRE intensity was reduced after 10 mM neomycin treatment for 24 h compared with the undamaged controls, and TMRE intensity in ARC-siRNA-transfected groups was reduced significantly more compared with the controls transfected with negative-siRNA. Data are shown as mean ± S.D. *p < 0.05. Scale bars = 20 μm.

The reduction of the mitochondrial transmembrane potential (MMP) is the main feature of cell apoptosis. The collapse of the MMP represents mitochondrial dysfunction and results in the opening of mitochondrial permeability transition pores, which allows apoptotic factors from the mitochondria to be released into the cytosol [14]. To further explore the detailed mechanism behind the increased neomycin-induced apoptosis of HEI-OC-1 cells after ARC inhibition, we took advantage of the TMRE kit to quantify the changes to the MMP in HEI-OC-1 cells. Immunohistochemistry and flow cytometry data showed that TMRE intensity was decreased after 10 mM neomycin treatment for 24 h compared with the undamaged controls (Figure 6A–6C), and after neomycin injury TMRE intensity was significantly decreased in the ARC-siRNA-transfected groups compared with the negative siRNA controls (Figure 6A–6C). This demonstrated that ARC expression might be important in maintaining the MMP of HEI-OC-1 cells and that ARC inhibition exacerbates the mitochondrial dysfunction, which leads to cell apoptosis.

### ARC inhibition leads to imbalances of oxidant factors and increases the reactive oxygen species levels after neomycin injury

**Figure 7:**
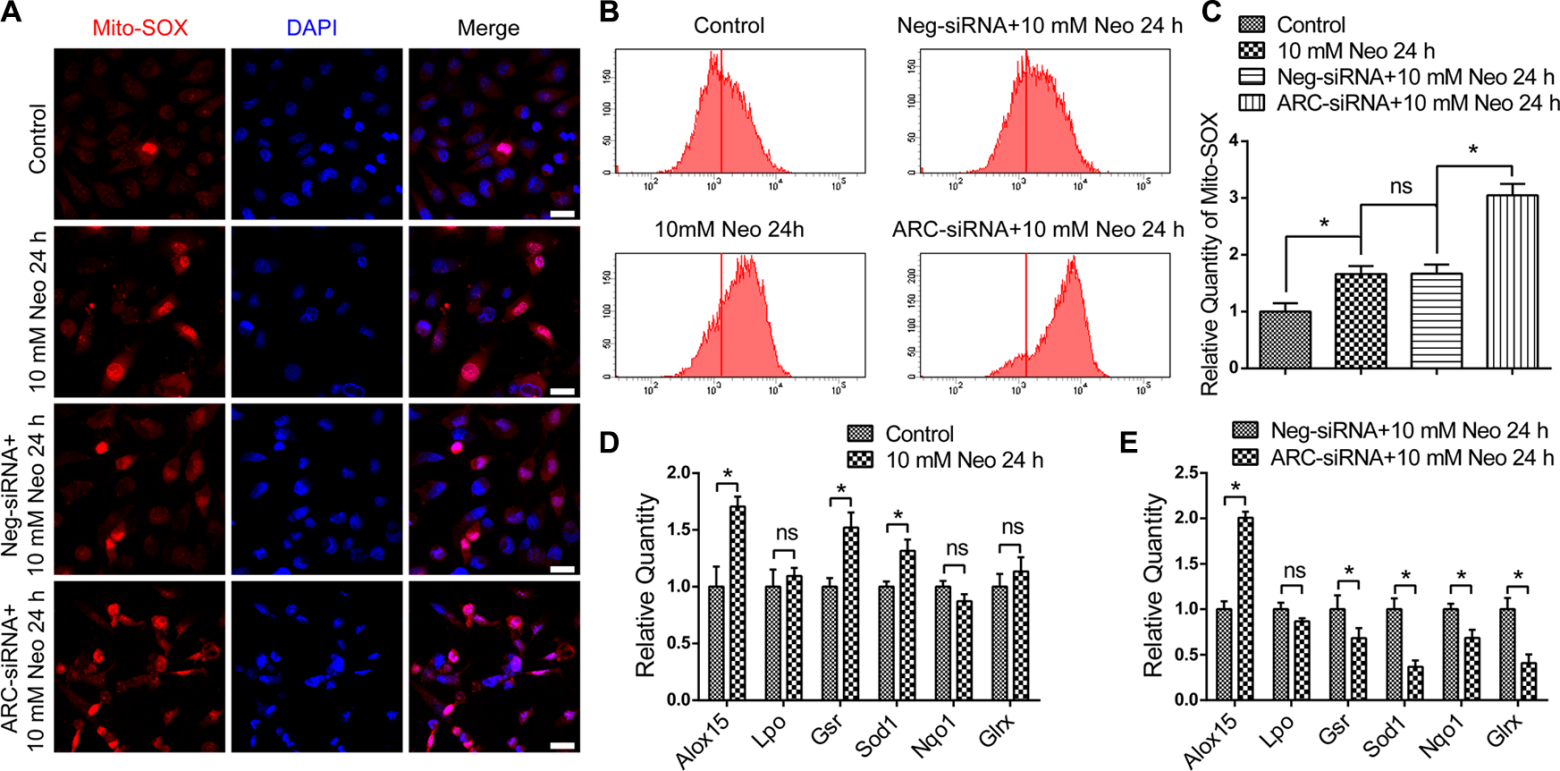
ARC inhibition with siRNA elevated the ROS and pro-oxidant factor levels and decreased antioxidant factor levels (**A**–**C**) The immunofluorescence and flow cytometry analysis showed that Mito-SOX intensity was increased after 10 mM neomycin treatment for 24 h compared with the undamaged controls, and Mito-SOX intensity in the ARC-siRNA-transfected groups was significantly increased compared with the negative-siRNA controls. (**D**) RT-qPCR showed that the expression of the pro-oxidant factor Alox15 and the antioxidant factors Gsr and Sod1 were increased after 10 mM neomycin treatment for 24 h compared with the undamaged controls. (**E**) RT-qPCR showed that the expression of the antioxidant factors Gsr, Sod1, Nqo1, and Glrx were significantly decreased, while the expression of the pro-oxidant factor Alox15 was significantly increased in the ARC-siRNA-transfected groups compared with the negative siRNA controls. Data are shown as mean ± S.D. *P < 0.05. Scale bars = 20 μm.

Accumulation of reactive oxidative species (ROS) in mitochondria is the main driving force of apoptosis, and ROS have been reported to play important roles in noise-induced and ototoxic drug-induced HC damage and hearing loss [5, 6, 15, 16]. We used Mito-SOX Red, a redox fluorophore that selectively detects mitochondrial superoxide, to evaluate mitochondrial ROS levels in HEI-OC-1 cells after 10 mM neomycin treatment for 24 h. Immunohistochemistry and flow cytometry results showed that the mitochondrial ROS level in HEI-OC-1 cells was increased after neomycin treatment compared with the undamaged controls (Figure 7A–7C), and ARC inhibition with siRNA significantly increased the mitochondrial ROS level in HEI-OC-1 cells compared with the negative-siRNA controls (Figure 7A–7C).

Cellular redox homeostasis depends on the antioxidant–pro-oxidant balance, and the increase in ROS levels might be caused by decreased antioxidant levels and/or increased pro-oxidant levels [16]. Here we analyzed the mRNA expression of six redox-related genes by qPCR. We found that the expression of most pro-oxidant factors and antioxidant factors was increased after neomycin treatment compared with undamaged controls (Figure 7D), while ARC inhibition by ARC-siRNA transfection significantly decreased the expression of the antioxidant factors Gsr, Sod1, Nqo1, and Glrx and significantly increased the expression of the pro-oxidant factor Alox15 (Figure 7E). Together, our data demonstrated that ARC inhibition decreased the expression of antioxidant genes, increased the expression of pro-oxidant genes, and increased the mitochondrial ROS levels in HEI-OC-1 cells after neomycin injury, which leads to apoptosis.

## DISCUSSION

Recent studies showed that ARC is specifically expressed in terminally differentiated cells such as neurons, skeleton muscle cells, and cardiac muscle cells [7]. Mammalian sensory HCs and supporting cells are also terminally differentiated cells, and both originate from inner ear sensory cell progenitors [17]. Thus we speculated that ARC would be expressed in both HCs and supporting cells, but our results showed that ARC was only expressed in cochlear HCs and not in supporting cells (Figure 1). A possible reason for this might be that cochlear supporting cells still have the ability – although only to a very limited extent –to re-enter the cell cycle, divide, and regenerate HCs after injury [18–24], and thus ARC might only be expressed in cells that have undergone non-reversible terminal mitosis.

Previous studies of ARC focused on cardiac muscle cells, and they all showed that ARC is an anti-apoptotic factor that is expressed in response to a wide range of stresses and insults. Because ARC is expressed in cochlear HCs and because HCs are highly susceptible to aminoglycoside-induced ototoxicity [25, 26], we assumed that ARC might have an important protective function against aminoglycoside-induced HC damage. In this study, we explored the role of ARC in neomycin-induced damage in hair-cell-like HEI-OC-1 cells, and we showed that ARC decreased rapidly in both cochlear HCs and HEI-OC-1 cells after neomycin injury (Figures 2 and 3) and that ARC inhibition dramatically increased cell death and apoptosis of HEI-OC-1 cells after neomycin injury (Figure 4, Supplementary Figure S6).

Previous studies reported that ARC protein levels decreased during the injury process through proteasome degradation and that the change in the ARC mRNA level was not significant [27, 28]. Here we found that both the protein level and the mRNA level of ARC decreased in a time and dose-dependent manner during neomycin treatment. The protein expression decreased more than the mRNA expression (Figure 3), which suggested that neomycin injury induced the downregulation of ARC in both a pre-translational and post-translational manner.

Apoptosis is primarily regulated by the activation of caspases through either intrinsic or extrinsic pathways [29], and aminoglycosides are considered to mainly initiate the intrinsic apoptotic pathway [30], Interestingly, our current study revealed that ARC is involved not only in the intrinsic apoptotic pathway, but it also has an effect on extrinsic apoptotic factors after neomycin injury. When ARC was inhibited, we observed significantly increased expression of both intrinsic and extrinsic pro-apoptotic genes – including Casp3, Casp2, Apaf-1, Fadd, and Casp8– and decreased expression of the anti-apoptotic gene Bcl-2 after neomycin injury, which suggests an anti-apoptotic role of ARC after neomycin injury (Figure 5).

Mitochondria play a very important role in cell metabolism, and aminoglycoside-induced apoptosis in HCs is closely related to mitochondrial dysfunction, including decreased MMP and increased ROS [4–6, 31–34]. Previous studies have shown that the accumulation of ROS triggers mitochondrial depolarization, initiates apoptosis, and changes mitochondrial membrane permeability [6, 16, 35, 36]. The change in mitochondrial membrane permeability will further lead to the loss of MMP, which results in the opening of mitochondrial permeability transition pores that enables the release of apoptotic factors from the mitochondria into the cytosol [30]. Here we demonstrated that ARC inhibition dramatically increased the mitochondrial ROS level and decreased the MMP of HEI-OC-1 cells after neomycin injury (Figures 6 and 7), suggesting that ARC inhibition exacerbated the mitochondrial dysfunction in HEI-OC-1 cells after neomycin injury.

The balance between pro-oxidant and antioxidant genes is extremely critical for the accumulation rate of ROS. In this study, we found that ARC inhibition significantly decreased the expression of several crucial antioxidant genes, including Gsr, Sod1, Nqo1, and Glrx, while it increased the expression of the pro-oxidative factor Alox15 (Figure 7). Together, all of these results suggest that ARC inhibition disrupts the balance between pro-oxidant and antioxidant genes and leads to elevated ROS levels, which further contribute to mitochondrial dysfunction and the release of apoptotic factors from the mitochondria that promotes the apoptosis of HEI-OC-1 cells after neomycin injury.

In summary, we provide the first report that ARC is specifically expressed in cochlear HCs and HC-like HEI-OC-1 cells and that the expression of ARC decreases after neomycin injury in both HCs and HEI-OC-1 cells. We also demonstrate that ARC inhibition disrupts the balance between pro-oxidant and antioxidant genes, which increases ROS accumulation and leads to the decrease of MMP and the release of apoptotic factors from the mitochondria and promotes apoptosis of HEI-OC-1 cells after neomycin injury. Our findings provide new insights for novel therapeutic strategies for preventing HC death after drug-induced ototoxicity.

## MATERIALS AND METHODS

### Culture of cochlear explants

Cochlear sensory epithelium was dissected from P7 mice and cultured as previously reported [5], and 0.5 mM neomycin (Sigma-Aldrich, St. Louis, MO, USA) was added to damage HCs. After neomycin was removed, the tissues were cultured in serum-free medium for an additional 24 h. The animal experiments were conducted in accordance with the guidelines of the Institutional Animal Care and Use Committee of Southeast University and were approved by the Committee on the Ethics of Animal Experiments of Southeast University.

### Cell culture and viability assay

The HEI-OC-1 cell line was kindly given by Dr. Federico Kalinec from the Auditory Cell Biology Laboratory of the David Geffen School of Medicine at UCLA in June, 2014. The cell line was tested and verified with RT-PCR and immunohistochemistry prior to use in our experiments, and we confirmed that this cell line still expressed various HC markers, including Myosin7a and Myosin6a, and could serve as a HC-like cell line.

HEI-OC-1 cells were maintained in DMEM medium supplemented with 10% FBS and 100 IU/ml penicillin (Sigma-Aldrich, St. Louis, USA) at 37°C with 5% CO_2_. The Cell Counting Kit (CCK-8; Protein Biotechnology, Beijing, China) was used to determine cell viability. Briefly, HEI-OC-1 cells were treated with 1 mM to 20 mM neomycin for 1 h to 24 h in 96-well plates, CCK-8 solution (10 μl/well) was added, and the plates were further incubated for 0.5 h at 37°C. Optical densities were determined on a microtiter plate reader (BIO-RAD, Hercules, USA) at 450 nm.

### Design and transfection of siRNA

ARC-siRNAs were generated by GenePharma (Shanghai, China) to downregulate the expression of ARC in HEI-OC-1 cells, and a nonsense sequence was designed as a negative-siRNA control. Cells were transfected with these siRNAs using Lipofectamine 2000 (Invitrogen, Life Technologies, Waltham, USA) following the manufacturer's instructions. Briefly, cells were plated in medium without antibiotics the day before transfection such that 40% to 50% confluence was achieved at the time of transfection. Cells were transfected with 200 nM ARC-siRNA or Negative-siRNA for 12 h and then exposed to 10 mM neomycin for 24 h. After neomycin was removed, cells were cultured in serum-free medium for additional 24 h, then collected for western blot, RT-qPCR, immunofluorescence, and flow cytometry assays.

### Immunofluorescence

Primary antibodies were anti-ARC (Santa Cruz Biotechnology Inc, Dallas, TX, USA, 1:500 dilution), rabbit polyclonal anti-cleaved-caspase-3 (Cell Signaling Technology Inc, Danvers, MA, USA, 1:500 dilution), polyclonal anti-myosin7a (Proteus Biosciences, Ramona, CA, USA, 1:1000 dilution), and goat polyclonal anti-SRγ(sex-determining region γ)-box 2 (Sox2) (Santa Cruz Biotechnology, Dallas, TX, USA, 1:400 dilution). Briefly, cells were fixed in 4% paraformaldehyde, and nonspecific binding sites were blocked for 1 h in 0.3% Triton X-100 and 10% (v/v) heat-inactivated normal serum in PBS (PBT1). Samples were then incubated overnight at 4°C in PBT1 with primary antibodies. After unbound antibodies were removed, samples were incubated with the corresponding secondary antibodies conjugated with tetramethylrhodamine (TRITC), fluorescein isothiocyanate, or Cy5 (Abcam, Cambridge, UK). Counterstaining with DAPI (Sigma-Aldrich, St. Louis, MO, USA) allowed visualization of the cell nuclei. Specimens were examined by confocal fluorescence microscopy (Leica SP5, Heidelberg, Germany). Negative control experiments were performed as above by omitting the primary antibodies.

The TUNEL Kit (Roche, Indianapolis, IN, USA) was used to detect apoptotic cells according to the manufacturer's instructions. TMRE (Sigma-Aldrich, St. Louis, USA) was used to measure the MMP, and Mito-SOX Red (Life Technologies, Waltham, USA) was used to detect ROS. HEI-OC-1 cells were exposed to 10 mM neomycin for 24 h, cultured in serum-free medium for an additional 24 h after neomycin was removed, washed with PBS, and incubated with TMRE or Mito-SOX Red for 10 min at 37°C. Cells were washed in prewarmed PBS and imaged by confocal microscopy (LSM700; Zeiss, Heidenheim, Germany).

### Quantitative real-time PCR

For quantitative real-time PCR, total RNA was extracted with TRIzol reagent (Protein Biotechnology, Beijing, China) and the integrity of all RNA samples was evaluated by OD260/280 measurements. cDNA was obtained using the RevertAid First Strand cDNA synthesis kit (Thermo Fisher Scientific, Waltham, USA) according to the manufacturer's instructions. Real-time PCR using SYBR Green (Roche, Basel, Switzerland) was carried out with a Biosystems CFX96 Real-Time PCR apparatus (BIO-RAD, Hercules, USA). The primer sequences are shown in Table 2. The specificity of the PCR amplification was confirmed by agarose gel electrophoresis. Thermal cycling conditions were 20 s at 95°C followed by 40 cycles of 15 s at 95°C and 1 min at 62°C, and a final extension of 20 s at 72°C. Melting curves were calculated directly after amplification. Gene expression was measured by semi-quantitative analysis, and GAPDH was used as the normalization control. Each analysis was performed in triplicate.

**Table 2:** PCR sequences used in the experiments

Gene	Forward sequence	Reverse sequence
β-actin	ACGGCCAGGTCATCACTATTG	AGGGGCCGGACTCATCGTA
Myosin7a	CTTTAACAAGCGTGGTGCCATC	GATTGCTGCGTTGATCTTCTCC
Myosin6a	TCAGAAGACATCAGGGAGAAGC	TGTTCTTCAGATTGCAGCCACC
Caspase-3	AATCATGCCATTTGCCCAGC	CTCAAGTGTGTAGGGGGAGG
Caspase-2	CTGACAGGAGGAGCAGGATTTT	CACCGAGAAGGGGAGACTTG
Apaf1	AGGGTGTGAGAGGAGTGTGT	ATCACCTCGATGGACTTGCC
Fadd	ACAATGTGGGGAGAGACTGG	CCCTTACCCGATCACTCAGG
Caspase-8	AGCCTATGCCACCTAGTGAT	GGAGAGCTGTAACCTGTCGC
Bcl-2	GGTGAACTGGGGGAGGATTG	AGAGCGATGTTGTCCACCAG
Alox15	TCGGGACTCGGAAGCAGAAT	CCCATCGGTAACAGGGGAAC
Lpo	GTTCCAGCCAACTCACACCA	CTCCCACCAGAACTTGCCTGT
Gsr	TGCACTTCCCGGTAGGAAAC	GATCGCAACTGGGGTGAGAA
Sod1	GGAGCAAGGTCGCTTACAGA	AGTGACAGCGTCCAAGCAAT
Nqo1	TCCGAAGCATTTCAGGGTCG	GGGCCAATACAATCAGGGCT
Glrx	AGTCTGGAAAGGTGGTCGTG	CCATTAGCATGGCTGGACGA
ARC(R)	CAGTGTAGGGGAACGCAAAT	CCGGTCAATGGTCTCCGATG
ARC(m)	GGACCACAAGCCCGACTC	GCACGTTGCCCATTTCTTCG
GADPH	GCAAGAGAGAGGCCCTCAG	TGTGAGGGAGATGCTCAGTG

### Western blot analysis

HEI-OC-1 cells were harvested and lysed with RIPA buffer (Protein Biotechnology, Beijing, China) containing a protease inhibitor cocktail (Sigma, St Louis, Missouri, USA) for 30 min at 4°C. The lysates were centrifuged at 12,000 × *g* for 10 min at 4°C, and protein concentrations were calculated using the BCA Protein Assay Kit (Protein Biotechnology, Beijing, China). Equal amounts of protein were loaded onto a 12% Tris-glycine SDS-PAGE gel and separated at 120 volts for 1.5–2 h. The protein was then transferred to a nitrocellulose membrane and blocked with 5% milk in TBST buffer. Immunoblotting was performed with anti-ARC rabbit polyclonal antibody (1:1000 dilution) and anti-cleaved-caspase-3 rabbit monoclonal antibody (1:500 dilution). An anti-β-actin mouse monoclonal antibody (Abcam, Cambridge, UK 1:5000 dilution) was used as a loading control. Peroxidase-conjugated goat anti-rabbit (or anti-mouse) immunoglobulin G (Abcam, Cambridge, UK) was used as the secondary antibody. The proteins were detected using a SuperSignal West Dura chemiluminescent substrate kit (Thermo Scientific, Waltham, USA) according to the manufacturer's instructions.

### Flow cytometry

Annexin V-FITC and propidium iodide (PI) (BD, San Jose, USA) were used for apoptosis analysis according to the manufacturer's instructions. Briefly, the cells were collected, washed twice with cold PBS, and then resuspended in 1× binding buffer at a concentration of 1 × 10^6^ cells/ml. A total volume of 5 μl Annexin V-FITC and 5 μl PI were added and gently mixed with 100 μl cells and incubated for 15 min at room temperature in the dark. A total volume of 400 μl 1× binding buffer was added to the tubes. For TMRE and Mito-SOX analysis, HEI-OC-1 cells were trypsinized, collected, and resuspended in prewarmed (37°C) solution containing TMRE or Mito-SOX for 10 min followed by washing with PBS. The samples were analyzed by flow cytometry (FACSCanto, BD, San Jose, USA) as soon as possible, and all tests were repeated at least three times.

### Cell counts and statistical analyses

To quantify HCs in the neomycin-treated samples, the entire cochlea was imaged using a 40× objective and the remaining Myosin7a-positive HCs were counted. A two-tailed, unpaired Student's *t*-test was performed when comparing two groups, and a one-way ANOVA followed by a Dunnett's multiple comparisons test was used when comparing more than two groups. A *p*-value < 0.05 was considered statistically significant.

## SUPPLEMENTARY MATERIAL FIGURES


