# Supplementary material for: Inhibition of ARC decreases the survival of HEI-OC-1 cells after neomycin damage in vitro
Source: Oncotarget. 2016 Aug 20;7(41):66647–59. doi: 10.18632/oncotarget.11336 (PMC5341827; doi:10.18632/oncotarget.11336)
Supplement: Supplementary file 1 [file oncotarget-07-66647-s001.pdf]

Inhibition of ARC decreases the survival of HEI-OC-1 cells after neomycin damage *in vitro*

Supplementary Materials

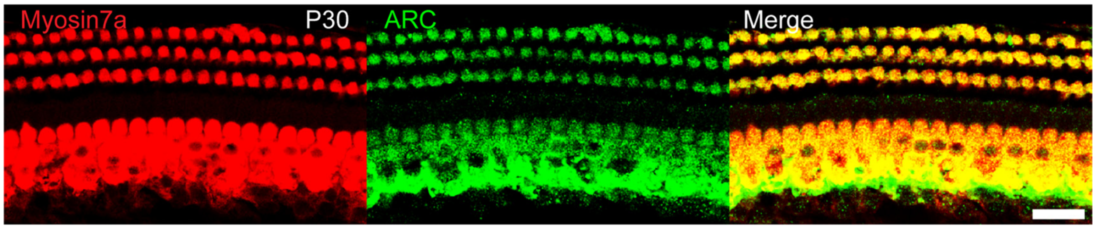

Supplementary Figure S1: Immunofluorescence staining showed that ARC was expressed in the P30 mouse cochlear HCs. Scale bars = 20 µm.

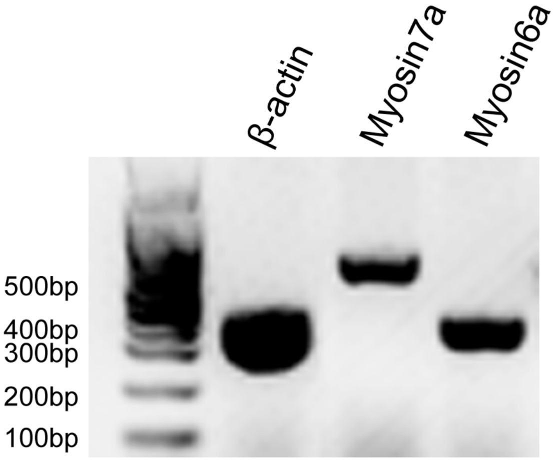

Supplementary Figure S2: RT-PCR showed that HEI-OC-1 cells expressed the HC markers Myosin 7a and Myosin 6a.

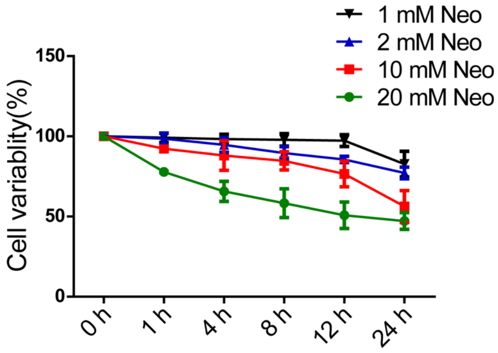

Supplementary Figure S3: The viability of HEI-OC-1 cells decreased gradually with both increasing neomycin doses and exposure time. Cell viability was detected by CCK-8 after HEI-OC-1 cells were treated with 1 mM to 20 mM neomycin for 1 h to 24 h in 96-well plates. Each condition was performed in triplicate. Data are shown as mean ± S.D. *$p < 0.05$.

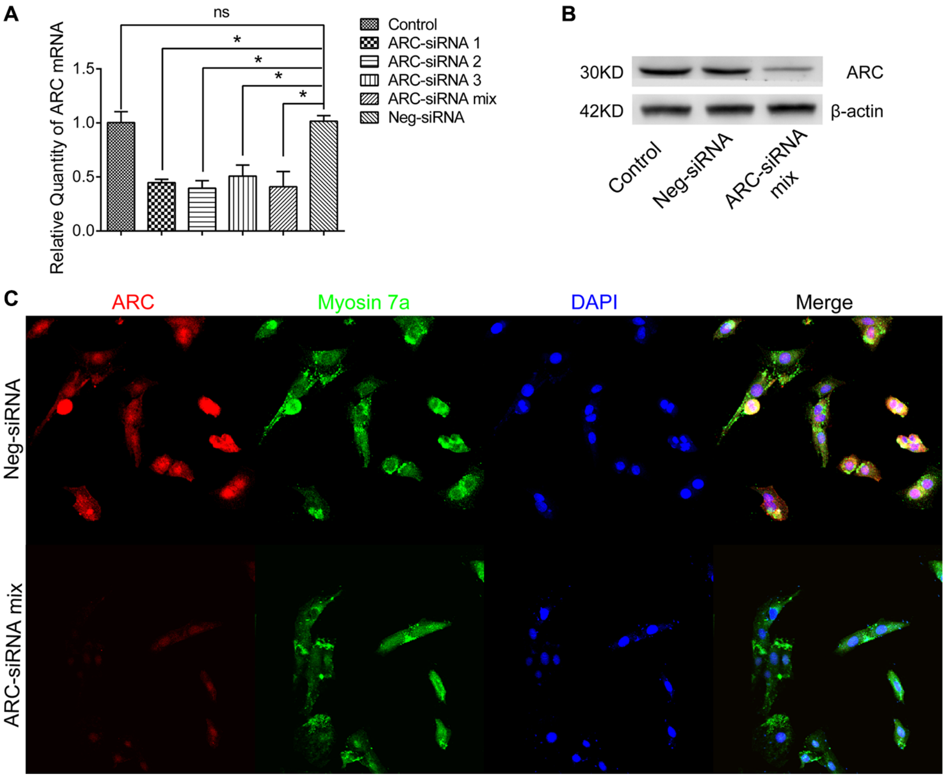

Supplementary Figure S4: ARC was inhibited by transfection with ARC-siRNA. (**A**) The mRNA levels of ARC decreased significantly after transfection with each ARC-siRNA and with the mixture of all three ARC-siRNAs. (**B**) The protein levels of ARC decreased significantly after transfection with the ARC-siRNA mixture. (**C**) Immunofluorescence staining showed that ARC expression was significantly decreased after transfection with the ARC-siRNA mixture. Data are shown as mean ± S.D. *$p < 0.05$. Scale bars = 20 μm.

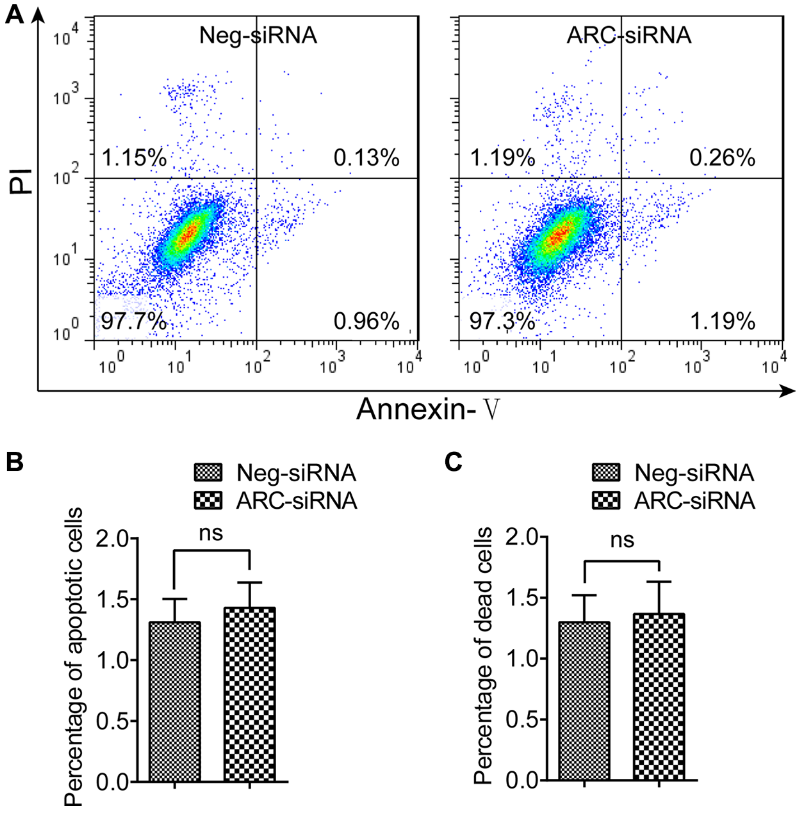

Supplementary Figure S5: Flow cytometry showed that there was no significant difference in the percentages of dead and apoptotic cells when ARC was inhibited without neomycin damage. Data are shown as mean ± S.D. *$p < 0.05$.

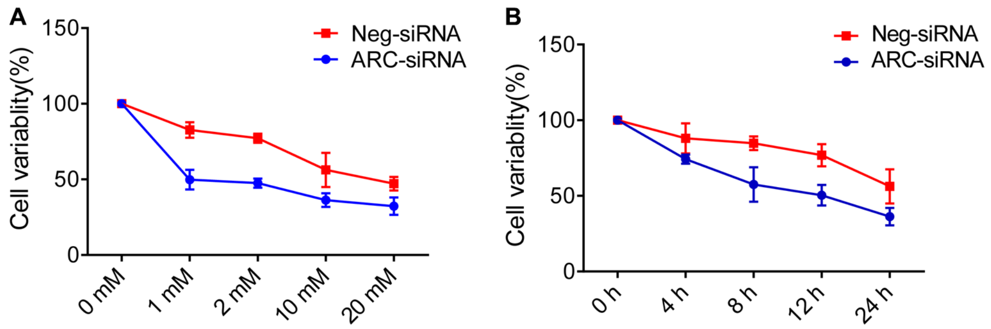

Supplementary Figure S6: The viability of HEI-OC-1 cells decreased significantly faster in the ARC-siRNA transfected groups when the dose and treatment time of neomycin increased. (**A**) HEI-OC-1 cells were transfected with ARC-siRNA or negative-siRNA and treated with 1 mM to 20 mM Neomycin for 24 h to determine the dose response curve. (**B**) HEI-OC-1 cells were treated with 10 mM neomycin for 1 h to 24 h to determine the temporal response curve. Cell viability was detected by CCK-8. Each condition was performed in triplicate. Data are shown as mean ± S.D. *$p < 0.05$.
